# Experimental data of designing an optimal system for storage, collection and transfer of household waste in the GIS environment: A case study of Tehran, district 22, Iran

**DOI:** 10.1016/j.dib.2018.06.064

**Published:** 2018-06-26

**Authors:** Mohammad Hadi Dehghani, Maral Fanaei Mosavi, Ali Asghar Ale-Sheikh, Zoha Heidarinejad, Mahmood Yousefi

**Affiliations:** a Department of Environmental Health Engineering, School of Public Health, Tehran University of Medical Sciences, Tehran, Iran; b Institute for Environmental Research, Center for Solid Waste Research, Tehran University of Medical Sciences, Tehran, Iran; c Islamic Azad University, Science and Research Branch, *Environment* energy, Department of Environmental Engineering, Tehran, Iran; d Department of Environmental Health Engineering, Faculty of Health, Hormozgan University of Medical Sciences, Bandar Abbas, Iran

**Keywords:** Household waste, Storage, Collection, Transfer, GIS

## Abstract

This study was conducted to correctly manage the system of storage, collection and transfer of wastes in district 22, Tehran. After reviewing existing methods, an optimal system was designed in the GIS environment and appropriate solutions were suggested. Analytical Hierarchy Process (AHP) method was used. After extracting result criteria, these criteria were provided to 15 experts and managers by means of a Delphi questionnaire. Screening of the criteria was done using the criterion importance graph; a necessary condition to apply criteria and sub-criteria, is having at least half the numerical value of each vertical and horizontal vector. The results of the study showed that the most important criterion associated with the selection of waste transfer station is "distance from residential houses" with a final weight of 0.341. "Suitable traffic conditions" and "lack of noise pollution" are the next important criteria with weights of 0.259 and 0.118, respectively. Finally, "non-destruction of recreational facilities" was chosen as the least important (weight of 0.03). Transfer in this district is also 100% mechanized. At the district level, there are 10 garbage trucks, of which 7 collect during night and 3 during day. Given per capita of the district, it takes about 10 min to collect each ton of waste. In general, in order to investigate and plan specific methods in the study district, using Geographic Information System, the location of reservoirs in residential and commercial districts has been determined and suggested with a coefficient of 0.75.

**Specifications Table**

**Table:** 

Subject area	Environmental Health
More specific subject area	Waste Management
Type of data	Table, Figures
Data format	Raw, Analyzed
Experimental factors	– Check the status of waste management district 22, Tehran – The optimal storage system for collecting and transporting waste was designed with GIS software. – Analytical Hierarchy Process (AHP) method was used to provide a solution.
Experimental features	The purpose of this study was to: 1) Design the transport routes and storage system; 2) Provide suitable solutions for improving municipal waste management.
Data source location	Islamic Azad University, Tehran, Iran.
Data accessibility	The data are available with this article

**Value of the data**

• The data of this study provides an efficient and optimal system for storing, collecting and transporting household wastes.
• The study of management elements of household waste that constitutes a major part of the waste of a community is effective in preventing ecological losses, such as water pollution and maintaining public health in a healthy environment.
• The waste collection process involves about 80% of the waste management costs. Therefore, to achieve public satisfaction and saving, designing an optimal collection system and determining the correct waste collection pathway can be conducted through organizing the collection.
• The results of the study showed that the distance from residential houses is the most important criterion in selecting the urban waste transfer station in terms of all aspects of environmental, human health and economic.
• The data of this study can be useful for solid waste management organizations and municipalities for the proper management of household wastes.

## 1 Data

**Fig. 1:**
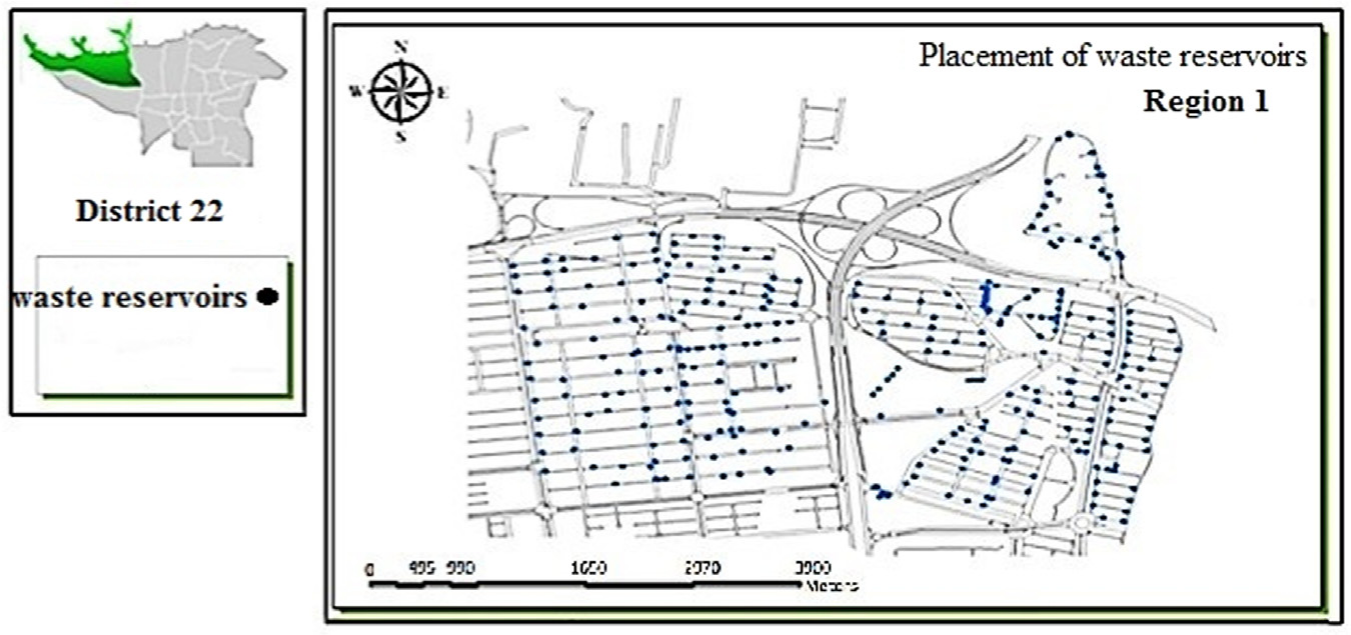
Optimal location of waste reservoirs in region 1.

**Fig. 2:**
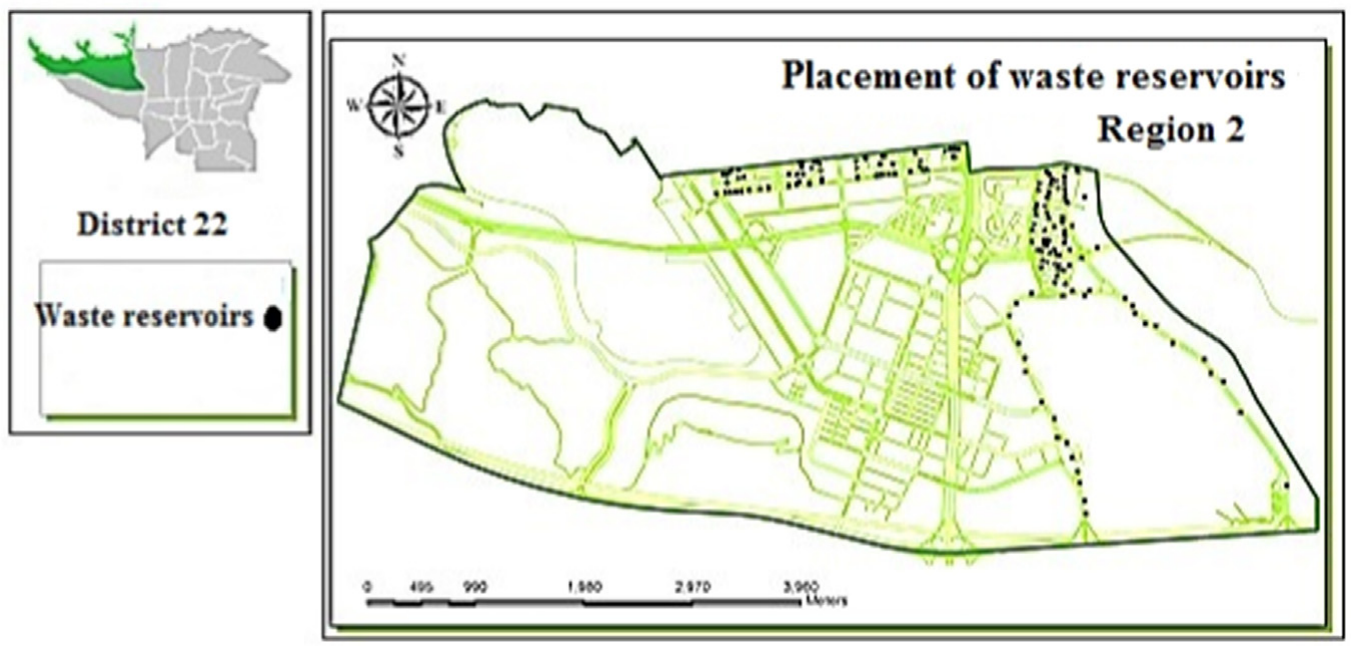
Optimal location of waste reservoirs in region 2.

**Fig. 3:**
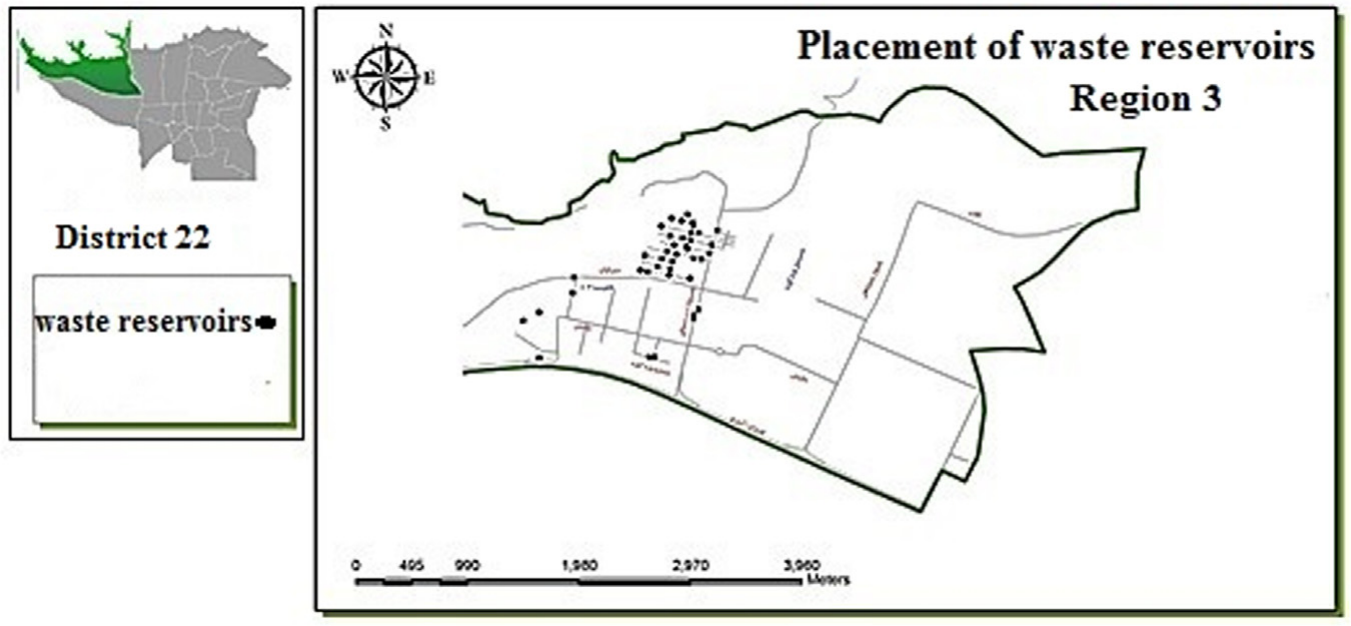
Optimal location of waste reservoirs in region 3.

**Fig. 4:**
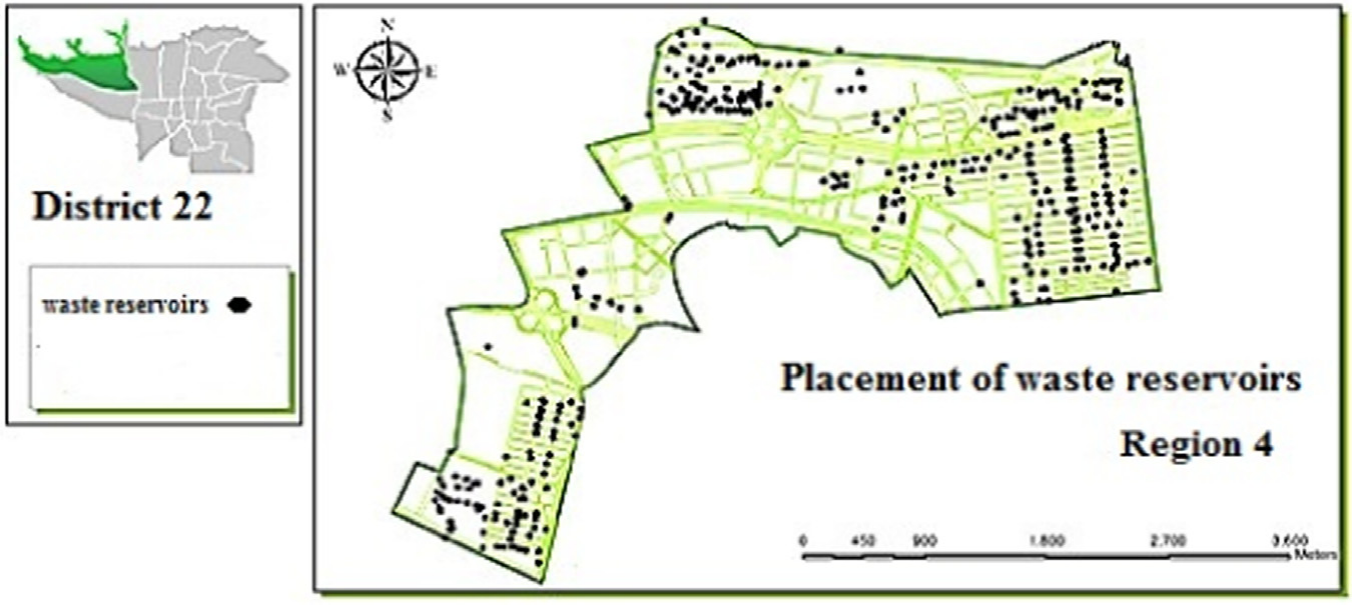
Optimal location of waste reservoirs in region 4.

Table 1 shows the status of waste management in the study district. Table 2 shows the duration of waste collection in different regions of the district. Fig. 1, Fig. 2, Fig. 3, Fig. 4 are related to the four regions of district 22, Tehran, and the waste reservoirs have been placed according to the criteria mentioned. Traffic routes of garbage trucks are displayed in different regions in Fig. 6, Fig. 7, Fig. 8. The classification of information layers are given in Fig. 9 to determine the optimal location of the waste transfer station. Table 3 shows the relative weights and inconsistency rates of the sub-criteria. The final weight of the optimal selection options for the urban waste transfer stations in different regions of the study district is indicated in Table 4. The prioritization of the optimal location criteria for urban waste transfer station is shown in Fig. 10 and the prioritization of the optimal location options for urban waste transfer station are shown in Fig. 11.

**Table 1:** The status of waste management in district 22 of Tehran municipality in different regions.

Region	Number of vehicles	Number of reservoir	Number of municipal solid waste workers	Rate of waste generation (kg)	Per capita waste generation (g/person/day)	Labor per capita (per thousand)
1	5	420	11	30,223	704	0.323
2	4	315	8	27,707	888	0.235
3	2	80	3	6927	888	0.088
4	6	570	12	34,026	727	0.352
Total	17	1385	34	98,883	802	1

**Table 2:** Duration of waste collection in district 22 of Tehran municipality.

Region	Pick up time (Hours)	Duration between Pick up time	Pick up time <total> (Hours)	Rate of waste generation (kg)	Average pick up timeper ton of waste (Hours)
1	1:20:06	3:45:10	5:05:16	30,223	0:10:25
2	1:41:19	3:17:08	4:58:27	27,707	0:08:59
3	1:09:06	1:58:27	3:07:33	6927	0:09:25
4	1:13:24	3:51:15	5:09:09	34,026	0:12:06
Total	5:38:20	12:52:00	18:12:55	98,883	00:10:023

**Fig. 5:**
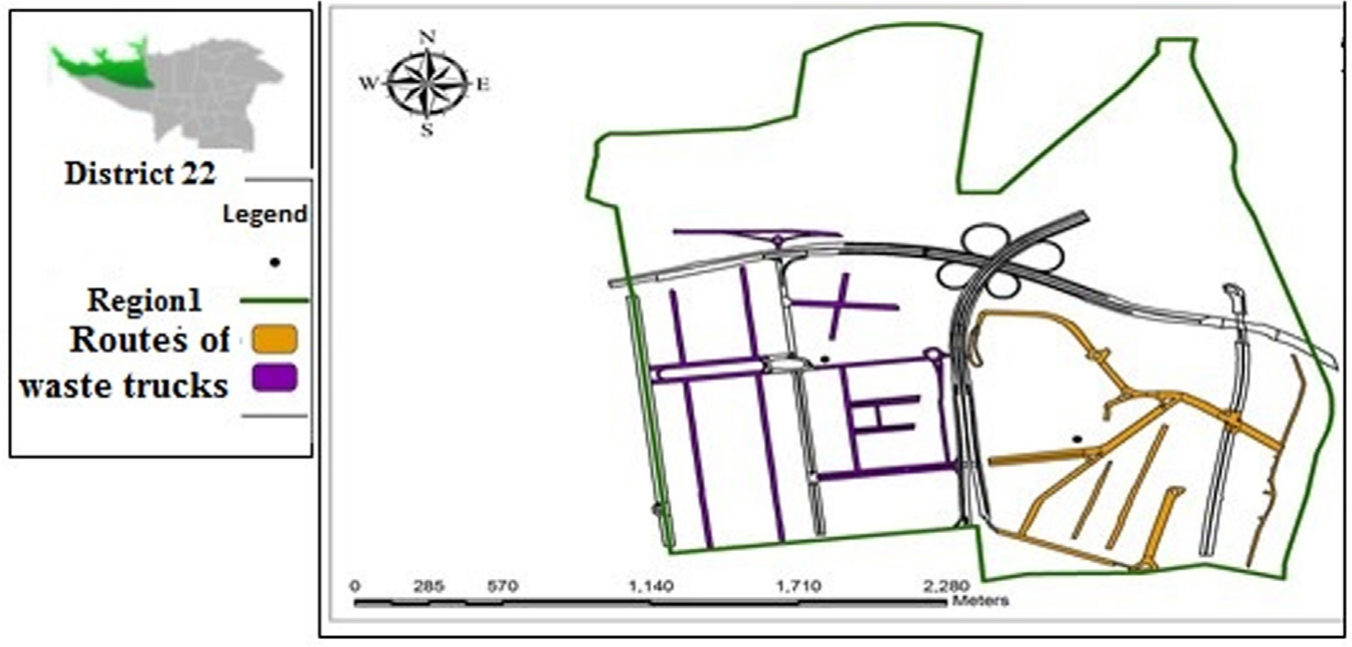
Traffic routes of waste trucks in region 1.

**Fig. 6:**
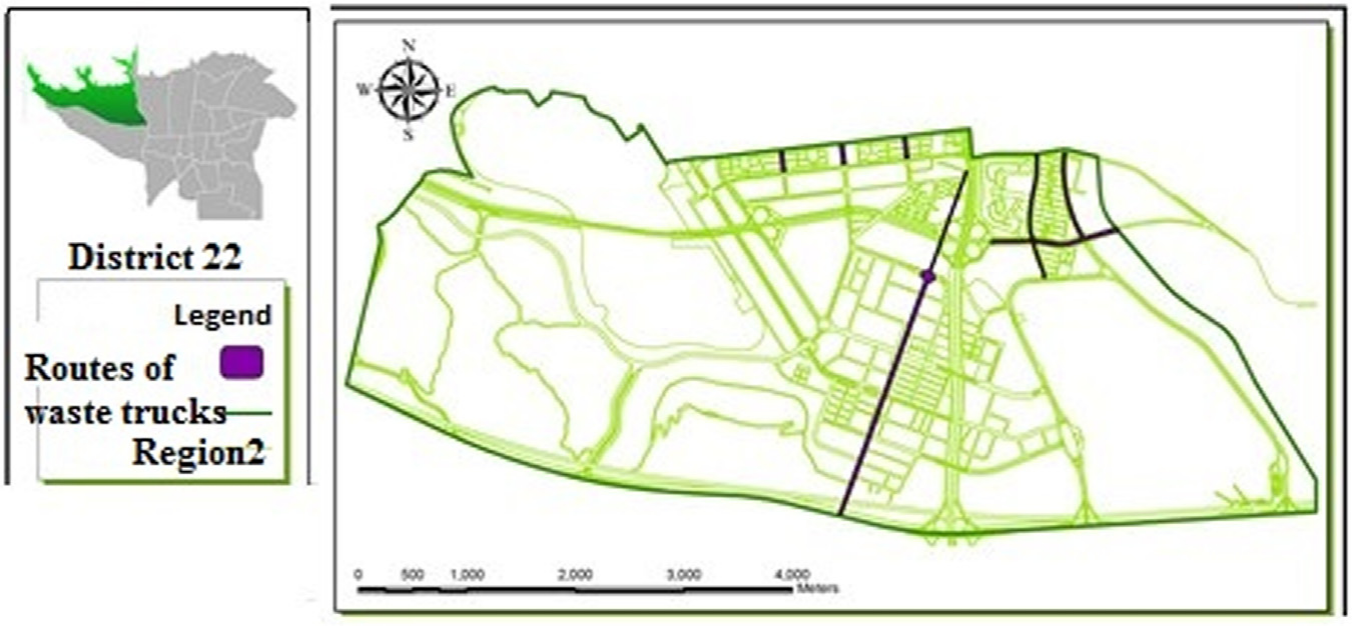
Traffic routes of waste trucks in region 2.

**Fig. 7:**
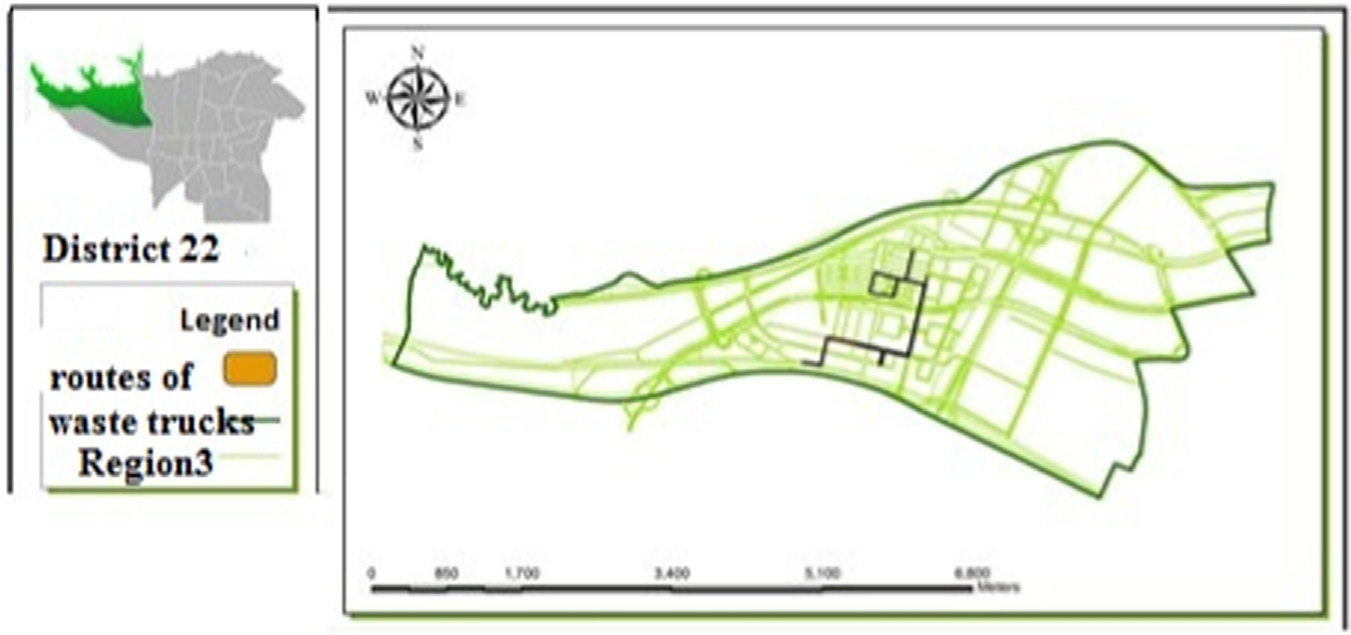
Traffic routes of waste trucks in region 3.

**Fig. 8:**
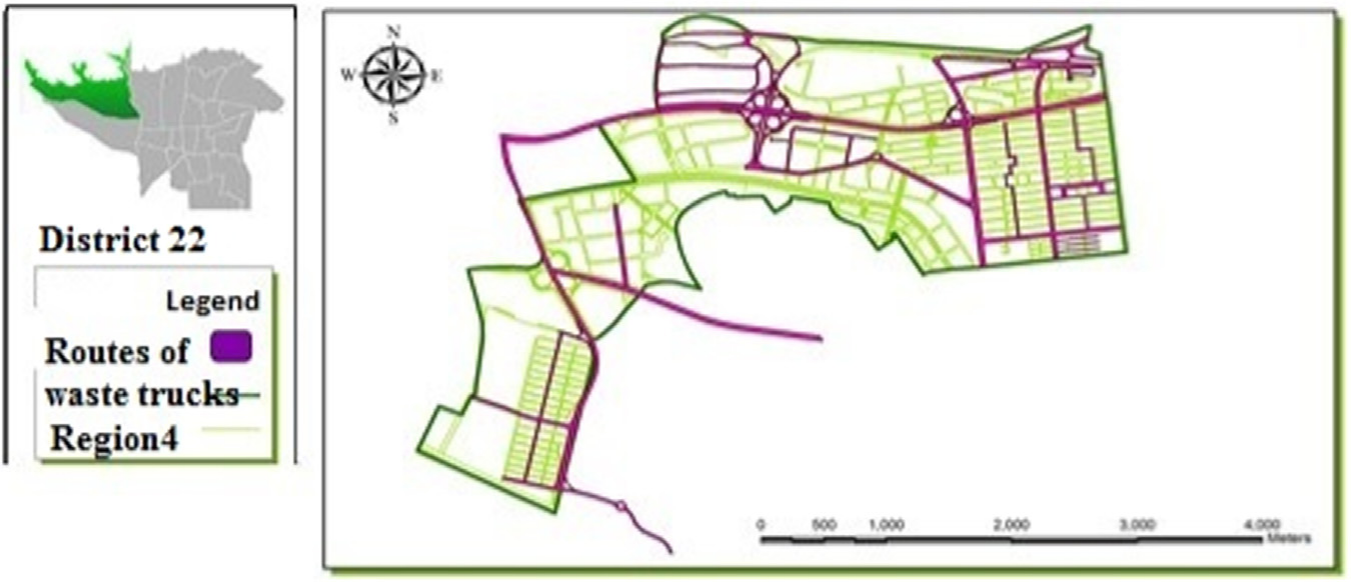
Traffic routes of waste trucks in region 4.

**Fig. 9:**
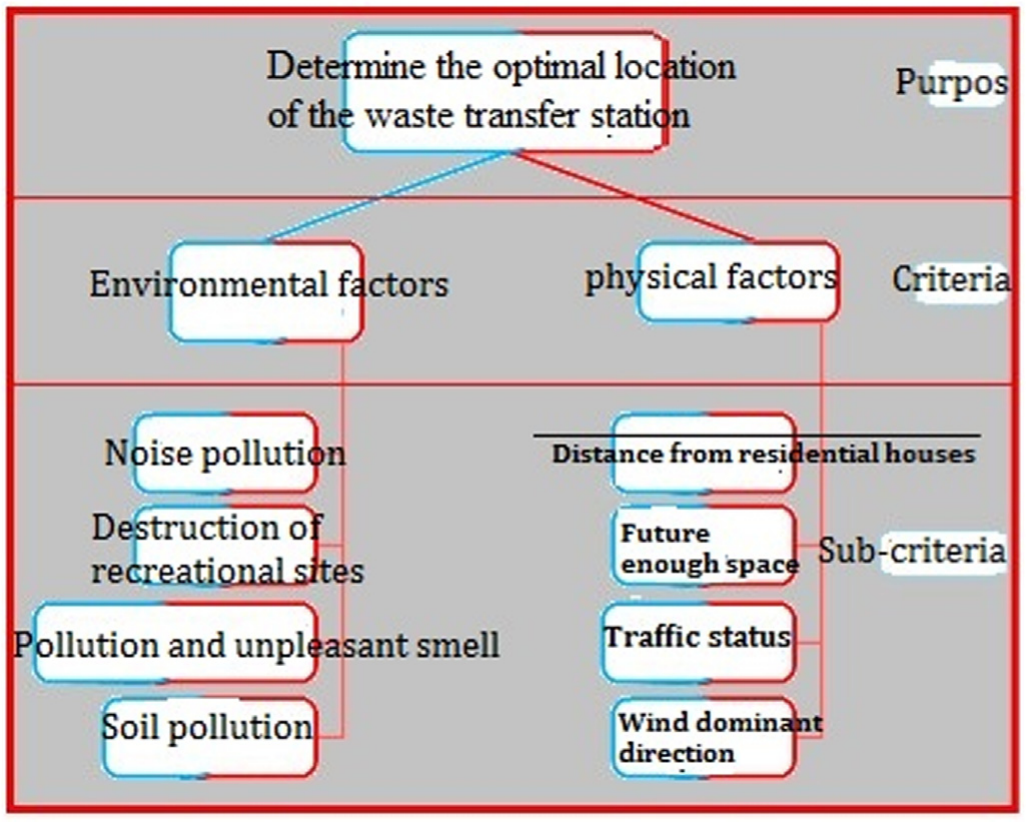
Classifying information layers (criteria and sub-criteria).

**Table 3:** Relative weight and inconsistency rate of the sub-criteria.

Sub-criteria	Relative weight	Inconsistency rate
Distance from residential houses	0.215	0.03
Traffic status	0.189	0.04
Noise pollution	0.144	0.05
Pollution and unpleasant smell	0.127	0.04
Wind dominant direction	0.105	0.05
Soil pollution	0.096	0.2
Future enough space	0.089	0.04
Destruction of recreational sites	0.035	0.08
Summary	1	0.35

**Table 4:** Final weight of the optimal selection options for the urban waste transfer station.

Options/criteria	1	2	3	4	Mean
D	0.334	0.332	0.31	0.388	0.341
F	0.265	0.254	0.281	0.237	0.259
E	0.104	0.108	0.136	0.126	0.118
B	0.097	0.077	0.103	0.093	0.093
C	0.078	0.064	0.062	0.057	0.065
A	0.059	0.059	0.048	0.041	0.052
H	0.032	0.057	0.039	0.039	0.042
G	0.031	0.049	0.021	0.019	0.03

**Fig. 10:**
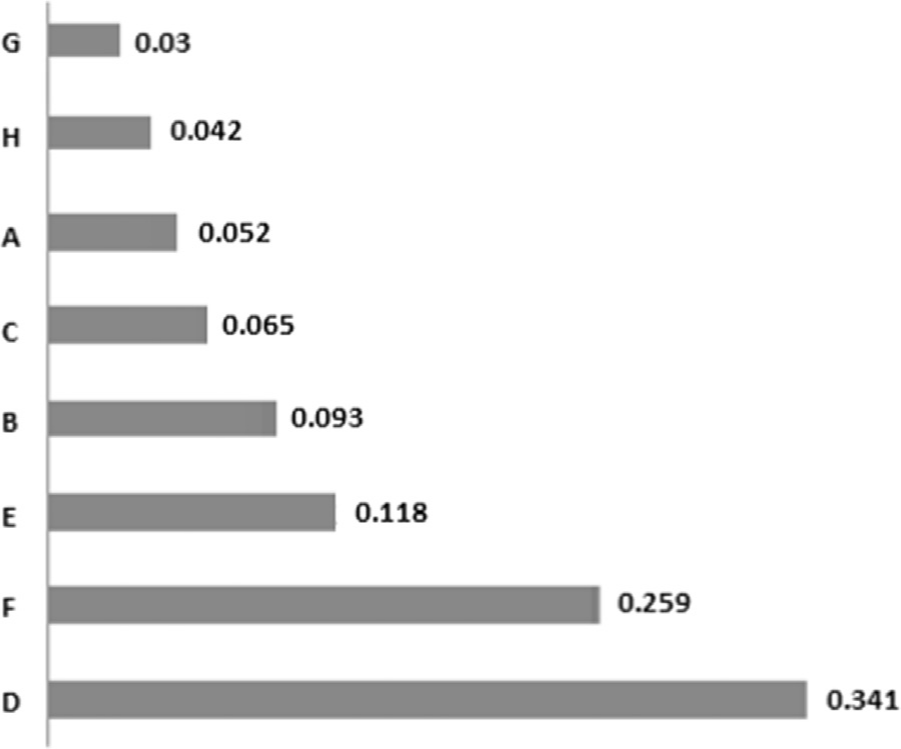
Prioritization of optimal locating options for the urban waste transfer station.

**Fig. 11:**
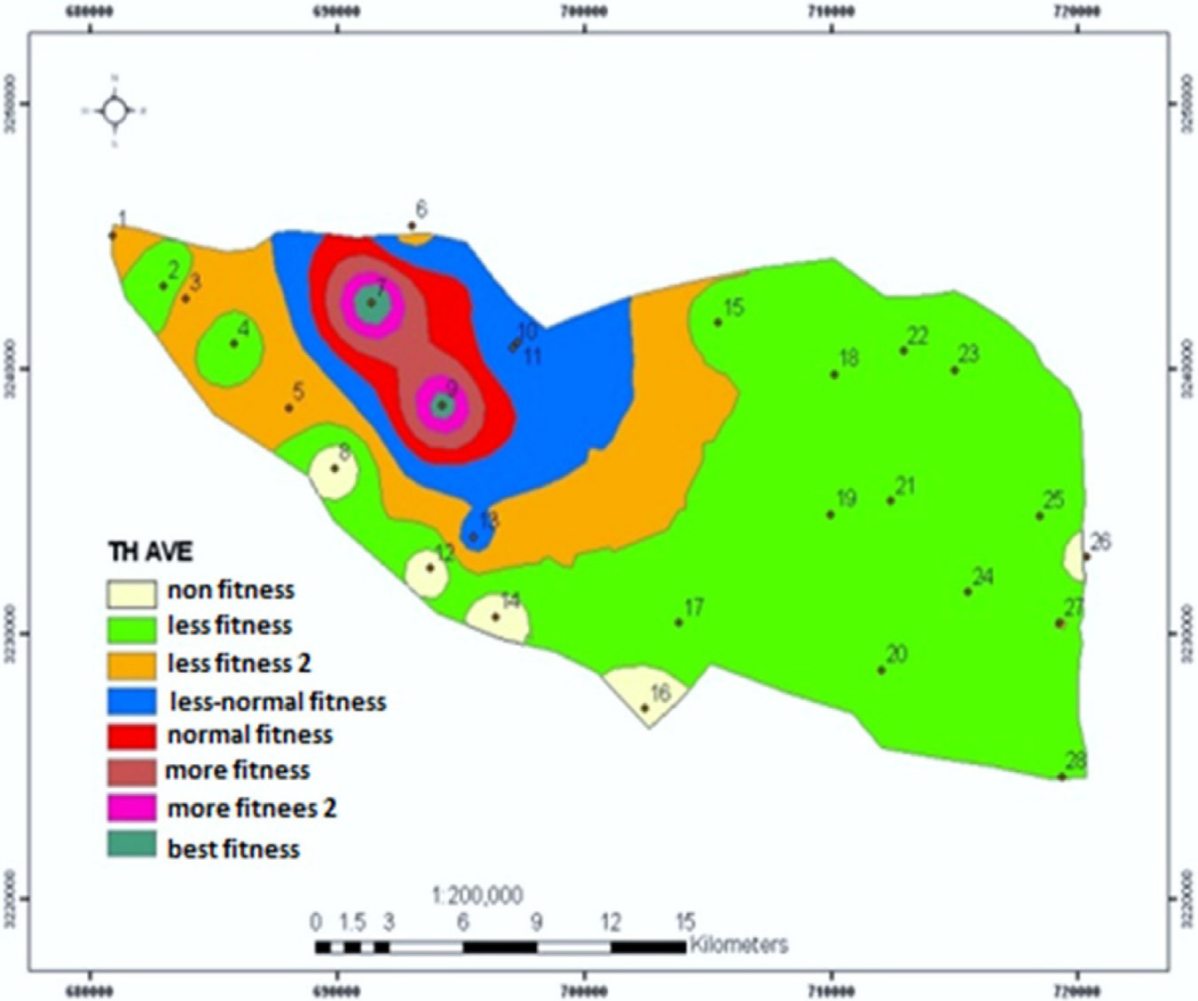
Prioritization of optimal locating criteria for the urban waste transfer station.

## 2 Experimental design, materials and methods

### 2.1 Study area description

District 22, Tehran municipality is located between east longitude 51 5′ 10′′ to 51 20′ 40′′, and north latitudes 35 32′ 16′′ to 35 57′ 19′′ in the northwestern part of Tehran and down the catchment basin of the Kan and Vardij Rivers. The district is about 54,000 ha accounting heights, with a maximum length and width of approximately 26 and 17 km. 8.4% of Tehran service limitation area belongs to district 22 and the approximate population of the district based on the preliminary results of 2006 census of population and housing is 138,970 people [1].

### 2.2 Material and methods

The present study was applied, descriptive and a case study. In this study, an optimal system in the GIS environment was designed and appropriate solutions were presented by reviewing existing methods of storing and collecting waste. Analytical Hierarchy Process (AHP) method was used to provide appropriate solutions [2], [3]. To achieve effective criteria in choosing the best solutions, firstly, effective methods for storing and collecting and transporting household wastes were gathered by reviewing the sources. After extracting the criteria used in the locating process and according to different criteria and functional aspects of the results, these criteria were provided to 15 experts and managers in the form of a modified Delphi questionnaire in order to judge and choose the appropriate criteria and sub-criteria in the process of collecting and transporting of waste (Fig. 5).

Prioritization of criteria and screening of sub-criteria were performed after analyzing the recommendations of experts and relevant specialists. The respondents were selected from among experts who had more than 5 years of experience working in the manufacturing, engineering, quality control, HSE and executive management of the municipality of district 22.

The respondents were asked about the importance of the criteria and sub-criteria identified, and they chose one of the 5° of importance (trivial with coefficient 1, low importance with coefficient 3, important with coefficient 7, very important with coefficient 9), where each showed the weight of itself. The number of options chosen for each degree of importance, presented the score of that degree of importance. In the next step, the amount of scores given for each criterion was calculated and the percentage of scores earned from the maximum obtainable weight were determined for each criterion and sub-criterion. The arithmetic mean score of the importance of each criterion was also calculated separately and considered for the final judgment. Screening of the criteria was conducted using the criterion significance graph, so that according to the relationship between the two components, the degree of importance of the criterion and the percentage importance of the criterion were calculated and the weighted mean of the degree of importance of each criteria is on the vertical axis, and some criteria were given in the intervention locating process which must be qualified sub-criteria to be selected. The necessary condition for the application of criteria and sub-criteria is to have at least half the numerical value of each vertical and horizontal vector. So, for the selection of the best criteria, we used criteria that were greater than half the numerical value of each vector. Finally, given the inconsistency of the weight, each criterion was calculated.

#### 2.2.1 Designing an optimal system for collection and transfer of waste in the study district

There is no specific guidelines for the placement of waste reservoirs and, depending on the needs of citizens or their objections, the location of the reservoirs has been determined and a bin is usually placed in each alley. But according to experts, for the design of the map, the distance of reservoirs is 100 m from each other and the width of the passage is considered to be at least 6 m [2], [4], [5], [6], [7], [8]. These issues were raised and executed due to taking into account the economic and cultural conditions as well as not to disturb by the reservoirs in the passageways for residents.

#### 2.2.2 Designing an optimal system of waste transfer routes in the study district

Traffic routes of the household waste trucks in district 22 are selected based on the fragmentation of the range and the shortest routes and include the main passages. Moreover, the arrangement of building blocks and the volume of waste generated are considered so that the shortest route was chosen.

#### 2.2.3 Determining an optimal waste storage system in district 22

For this purpose, the three following phases were done:

1) Extraction of the most important locating criteria for urban waste transfer stations;
2) Screening and selection of criteria by experts and specialists;
3) Weighting and prioritizing the criteria and sub-criteria.

Standard proposed criteria for each of the sub-criteria specified in the optimal mode with the least defined requirements were as follows:

A. The quantity and quality of leachate must be within the standard range to prevent soil contamination;
B. Unpleasant smell and air pollution from the transfer station must not move towards the residential districts;
C. The transfer station must not be in the wind dominant direction in relation to residential regions;
D. Distance of at least 1000 m from residential houses must be considered;
E. Environmental noise must be less than 55 dB per day, so it does not cause noise pollution;
F. The duration of transfer by trucks must not cause a disturbance in the traffic situation of the district;
G. Station must have enough space for the possibility to be expanded in the future for the current use;
H. Natural recreational sites of the district must not be affected by the location of the waste transfer station.
